# To determine the prognostic value of the albumin–bilirubin grade (ALBI) in patients underwent transarterial chemoembolization for unresectable hepatocellular carcinoma 

**Published:** 2019

**Authors:** Muhammad Ali Khalid, Inamullah Khan Achakzai, Farina M Hanif, Shoaib Ahmed, Zain Majid, Nasir Hassan Luck

**Affiliations:** *Department of Hepatogastroenterology, Sindh Institute of Urology and Transplantation, (SIUT), Karachi, Pakistan *

**Keywords:** Hepatocellular carcinoma, Unresectable disease, Transarterial chemoemobolization, ALBI grade, CTP score, MELD score

## Abstract

**Aim:**

We aimed at determining the prognostic value of the albumin–bilirubin grade (ALBI) in patients undergoing transarterial Chemoembolization for unresectable Hepatocellular carcinoma.

**Background:**

Various noninvasive liver reserve markers are used to predict the severity of liver injury. The role and probability of these markers in predicting the prognosis of patients with hepatocellular carcinoma (HCC) is still unknown.

**Methods:**

Patients who underwent TACE from 2013 to 2017 were included. Patient’s age, gender, cause of cirrhosis, ALBI Grade along with the site, size and number of tumors were recorded. Radiological response to TACE was assessed by CT scan at 1 and 3 months after the procedure, respectively. Survival assessment was performed and all patients were assessed for survival until the last follow-up.

**Results:**

A total of 71 patients were included. Majority of them were male (80.3 %). The mean tumor size of 6 ± 3.9 cm. Majority of patients (54.9 %) had a single lesion and it was mostly localized to the right lobe (60.5 %). The most common cause of chronic liver disease was HCV (65.3%). Median Child class score (CTP) and MELD score were 7 and 10, respectively. Ascites was treated prior to TACE in 12 patients (16.9 %).

Mean ALBI score in the study population was -1.59 ± 0.69, with the majority (49. 2 %) falling in grade 2. The mean duration of survival at the last follow up was of 12.1 ± 12.14 months (1- 49).

Univariate analysis showed serum albumin (p = 0.003), serum bilirubin (p = 0.018), CTP score (p = 0.019), ALBI grade (p = 0.001) and presence of varices (p = 0.04) to be the main predictors of 6 months survival after TACE. On Cox analysis, only ALBI score (p = 0.038) showed statistical significant association.

**Conclusion:**

ALBI grade may serve as a surrogate marker in predicting the prognosis of HCC patients undergoing Transarterial Chemoembolization.

## Introduction

 Hepatocellular carcinoma (HCC) accounts for 70%-85% of major liver cancer burden globally (1). It is the sixth most common malignancy and the third most cause of cancer-related mortality worldwide (2) The incidence of HCC is higher in Southeast Asia and sub-Saharan Africa regions. In Pakistan, the prevalence of HCC varies from 3.7%-16% of malignant tumors, with about 87% of HCC being mainly due to viral hepatitis, that is Hepatitis C (68%) or B related cirrhosis (22%). The incidence of HCC in Pakistan is 7.6 per 100,000 persons per year for males and 2.8 per 100,000 persons per year for females (3,4).

HCC mostly develops on a milieu of chronic liver disease or cirrhosis (5,6). As a consequence, various degrees of liver functional insufficiency are typically present at the point of cancer identification. For patients with early stage and well preserved liver function, surgical resection and liver transplantation are usually suggested (7). As a result, in unresectable HCC, radiofrequency ablation (RFA) and transarterial chemoembolization (TACE) are the potential palliative modalities (8). Transarterial chemoembolization is commonly acknowledged as a palliative treatment option and improves survival in unresectable HCC (9, 10).

Parameters assessing liver function have been integrated into majority of the staging systems (11-14). However, the clinical management of HCC primarily depends upon its clinical stage of the disease (11). Nevertheless, the precision of the current ongoing staging systems in predicting the prognosis and guiding management is not satisfactory. It was recommended that the unsatisfactory prognostic performance of the staging systems could somewhat be explained by the inadequate liver function gauging system (14).

Among patients with chronic liver diseases of diverse etiologies as well as HCC, the Child–Pugh (C-P) class is generally used to approximate prognosis and disease severity (15). However in C-P class A patients, with apparently normal liver functions, prognosis varies widely (16,17) In addition, some of the variables in the C-P grade are interconnected (e.g., ascites and serum albumin levels), and the grading of ascites and encephalopathy can be highly subjective (17-19). Hence, the shortcomings of the C-P grading system leaves an area for the improvement of another liver function estimation systems.

 In patients with chronic liver diseases, various noninvasive liver reserve markers like the model for end-stage liver disease (MELD), Lok index, cirrhosis discriminant index (CDS), fibrosis index based on 4 factors (FIB-4) and aspartate aminotransferase-to-platelet ratio (APRI), have been suggested to assess the degree of functional liver reserve (20). Recently, the albumin-bilirubin (ALBI) grade was introduced as a prognostic marker which was solely based on the serum albumin and bilirubin level (21).

 Selecting the most favorable surrogate marker for these patients is contentious. Given all these choices, the role of these markers and their accuracy in foretelling the outcome of HCC patients remains largely uncertain. Thus the aim of this study was to evaluate the prognostic value of the new liver function assessment tool, the albumin–bilirubin (ALBI) grade, in patients with hepatocellular carcinoma undergoing TACE. 

## Methods

**Definitions**

The primary C-P score is divided into three sub classes (A= 5–6 points, B = 7–9 points, and C= 10–15 points). 

The ALBI grade was calculated using the following formula: 0.66 × log (bilirubin mg/dl)_0.085 × (albumin g/dl). The cut-off points for ALBI grades 1–3 were: grade 1, less than -2.60; grade 2, -2.60 to -1.39; and grade 3, more than -1.39 (21).

**Patient selection**

All patients diagnosed as HCC according to the American Association Study of Liver Disease (AASLD) criteria 15 and found eligible for TACE were included in this study. The study period was of five years, i.e from September 2013 to December 2017. Those patients who presented within 6 months of any previous intervention such as RFA or surgical liver resection for HCC were excluded from this study.

**Inclusion criteria**

Patients of either sex, of all ages, diagnosed with non-resectable and non-ablatable HCC were enrolled in this study. We defined cases of HCC as non-resectable when any one or more of the following conditions were present: severe comorbidity that precluded the administration of general anesthesia; liver dysfunction and/or portal hypertension that contraindicated parenchyma loss during radical tumor resection. Ablation therapy was not indicated when the maximum diameter of the tumor was >5 cm, when the tumor was in close proximity to major vascular or biliary structures, or if there was multifocal disease.

**Methods**

This was a retrospective observational study. Approval was obtained from the Ethical Review Committee (ERC) of Sindh Institute of Urology and Transplantation, Karachi, Pakistan (SIUT), with informed consent being obtained from the patients before enrollment. A total of 71 patients were included in this study. The TACE procedure was performed in the Radiology Department of our institute with the procedure involving injection of a chemotherapeutic agent (doxorubicin) mixed with lipoidal into selectively or super selectively catheterized branches of the arteries feeding the tumor followed by injection of gelfoam particles to reinforce the effect of treatment. After the procedure, the patients were shifted to the Gastroenterology Ward for observation. A structured proforma was used to collect data and included demographics (age, gender), clinical (etiology), laboratory parameters [serum bilirubin, albumin, creatinine, international normalized ratio (INR), and alphafetoprotein (AFP)] and imaging (number of lesions, size, and lobe involved), the Child-Turcotte-Pugh (CTP) score and the Model of end-stage liver disease (MELD) score (22). At the end of 6 weeks, a computerized tomography (CT) scan of the abdomen was performed as per the TACE protocol. Response of TACE was evaluated according to the modified Response Evaluation Criteria in Solid Tumors (mRECIST) criteria. Inquiries were made through telephone calls to determine the patient's survival status.

**Statistical analysis:**

Data were statistically analyzed using Statistical Package for the Social Sciences (SPSS) software version 20.0 (Chicago, IL, USA). Frequencies and percentages were computed for different categorical variables such as gender and cause of HCC. Mean and standard deviation were computed for age. We employed the two-sided Fisher’s exact test to analyze the dichotomous variables before and after TACE. Univariate analysis and multivariate analysis were also performed. A *P*-value of <0.05 was considered statistically significant. Survival analysis was done using the Kaplan- Meier estimates, with comparisons generated using the log rank test. 

## Results

A total of 71 patients were included in our study. Patients demographic and tumor characteristics are shown in Table 1. Majority of our patients were male i.e 57 patients (80.3 %) and a mean age of 51.9 ± 12.1 years (18 – 76 years) was observed. The mean tumor size was of 6 ± 3.9 cm. Majority of the patients (54.9 %) had a single lesion and these were mostly localized to the right lobe of the liver (60.5 %). The most common cause of chronic liver disease was HCV (65.3%) while a cryptogenic cause was documented in 10 patients (14.1%). The median Child class score (CTP) and MELD score were 7 and 10, respectively. 

**Table 1:** Demographics and tumor characteristics of study population (n = 71)

Variables		p- value
Age	51.9 ± 12.1 (18 - 66)	0.215
Male	57 (80.3 %)	0.37
Serum Albumin	2.8 ± 0.6 (1.4- 4.8)	0.003
Bilirubin	1.4 ± 1.1 (0.3- 6.3)	0.018
INR	1.2 ± 0.2 (1- 1.8)	0.70
MELD, median	10	0.21
CTP score	7	0.019
A	33 (46.5%)	
B	35 (49.3%)	
C	3 (4.2%)	
Cause of liver disease		
HCV	45 (63.4%)	
HBV	8 (11.3%)	
HBV+HCV	5 (7.0%)	
HBV+HDV	1 (1.4%)	
HBV+HCV+HDV	2 (2.8%)	
Cryptogenic	10 (14.1 %)	
ALBI grade		0.001
-2.60 and above	6 (8.4 %)	
-2.59 to --1.39	35 (49.2 %)	
-1.38 to +1	30 (42.2 %)	
Varices, n (%)		0.04
Present	30 (55.6 %)	
Absence	24 (44.4 %)	
Tumor size, mean	6.0 ± 3.9 (1.6- 18)	0.84
Less than 5 cm	42	
More than 5 cm	29	
Lesions		
Single	39 (54.9%)	
Two or more	32 (45.1)	
Lobes		0.48
Right	43 (60.6 %	
Left	17 (23.9 %)	
Both	11 (15.5 %)	
TACE session		
1	43 (60.6%)	
2	17 (23.9 %)	
3 or more	11 (15.5 %)	
Recurrence	47 (66.2 %)	0.198
Survival		
Duration of survival	12.1 ± 12.14 (1- 49)	

**Table 2:** Association btw ALBI grade and survival

3 months	55 (77.5%)	0.010
6 months	39 (54.9%)	0.001
12 months	31 (43.7%)	0.00

**Table 3:** Multivariate Analysis on association of factors with 6 months Survival

Variables	Hazard Ratio	p- value
CTP score	0.64	0.09
ALBI grade	3.06	0.038
Varices,	0.97	0.64

Fifty four patients had consented for an EGD and esophageal varices were seen in 30 (55.6 %) patients. Ascites was treated prior to TACE in 12 of these patients (16.9 %).

The mean ALBI score in the study population was -1.59 ± 0.69, with majority ( 49. 2 %) being in ALBI grade 2.While the mean duration of survival at the last follow up was 12.1 ± 12.14 months (1- 49).

Univariate analysis showed that serum albumin (p= 0.003), serum bilirubin (p= 0.018), CTP score (p= 0.019), ALBI grade (p= 0.001) and the presence of varices (p= 0.04) were significant predictors of 6 months survival post TACE. On Cox regression analysis, only ALBI score (p= 0.038) showed statistical significant association.

## Discussion

TACE has a considerable survival benefit in the management of non-resectable HCC. Llovet *et al.* (9) reported a 2 year survival in 63% of the patient while Lo *et al. *(10) showed a 26% three-year survival advantage in patients who underwent TACE. A recent meta-analysis also supported the advantage of chemoembolization in the selected patients (23). For these reasons, TACE has been established as a treatment of choice in patients with unresectable HCC. Chronic hepatitis C appears to be the major risk factor for the development of HCC which is consistent with our study population, where HCV has accounted for 63.7% of patients.

In our study we validated the prognostic ability of ALBI grade in patients with HCC who underwent TACE. We further discovered that ALBI Grade has gained a superior prognostic differentiating efficacy than that of C-P in HCC patients, which augmented the results by Johnson *et al*. (21). In the modern surgical era, reducing morbidity and mortality are still major concerns, numerous studies have recommended that precise evaluation of liver function reserve is indispensable for prognosticating the occurrence of morbidities and mortalities (24). The C-P score was usually calculated to estimate the risks (24). Our study shows that the ALBI score, could also be used to estimate the post TACE morbidities.

The survival outcome, morbidity, mortality and the treatment options in HCC are not only reliant on the tumor stage, but also on liver functional reserve compared with other solid tumors (24). The C-P score, which was originally evolved for patients with cirrhosis rather than HCC, is the most extensively acknowledged liver function assessment tool and thus is included in a number of tumor staging systems including BCLC12 and the CLIP (25). Several fundamental flaws of the C-P class exist. First, clinical assessment of ascites and hepatic encephalopathy can be highly biased. Presence or severity of ascites, by some practitioners, is determined by physical examination. Others may consider ascites only when it is detectable by radiological scanning. The tumor itself and the diuretic may also impact ascites. Encephalopathy may be equally difficult to grade, as many of the early symptoms may be shared by the HCC itself. Second, some of the indexes in the C-P class, such as ascites and serum albumin, are closely interconnected. In fact, the five items may not influence the clinical outcomes at an identical level.

Hiraoka *et al*. (26) compared the predictive value of ALBI grade and C-P score in 2584 Japanese HCC patients. The ALBI grade was found to be better for distinguishing patients with better hepatic function. In our study on univariate analysis the ALBI grade, CTP score and varices were closely related to overall survival of HCC patients but on multivariate Cox analysis, only ALBI grade was found to be statically significant and those patients with ALBI grade 2 and grade 3 had increased risk of mortality when compared to patients with ALBI grade 1.

The ALBI score has some advantages when compared to other prognosticating scores. It is the simplest liver function estimating score to date because it requires only two parameters, the serum bilirubin and albumin. These can be acquired from the routine liver function blood test, which are available in almost every medical setup both in the rural and urban areas. Moreover, the ALBI grade get rides of the subjective items such as hepatic encephalopathy. The objectivity and accuracy of the grading system are merited.

Our study had some limitations. This was a single center study with retrospective data collection. Hepatocellular carcinoma was diagnosed on the basis of CT scan as per AASLD criteria. Although rare, the possibility of mixed HCC and cholangiocarcinoma (CC) could not be entirely excluded. Second, this study was limited to HCC patients undergoing TACE. Hence the accuracy of ALBI grade in patients receiving other therapies required further studies to recognize.

Our results signify that the ALBI grade is the most precise prognostic model among the other noninvasive liver reserve markers as supported by Shu-Yein Ho *et al*. (27). The ALBI grade may provide as an objective, discriminatory and evidence-based method in assessing liver functional reserve. The ALBI grade is clinically more useful due to is higher prognostic power in HCC patients undergoing TACE. Further studies are now needed to authenticate the practicability of ALBI grade in diverse clinical scenarios.

## Conflict of interests

The authors declare that they have no conflict of interest.
